# Melanocyte Chitosan/Gelatin Composite Fabrication with Human Outer Root Sheath-Derived Cells to Produce Pigment

**DOI:** 10.1038/s41598-019-41611-5

**Published:** 2019-03-26

**Authors:** Xianyu Zhou, Yan Ma, Fei Liu, Chuan Gu, Xiuxia Wang, Huitang Xia, Guangdong Zhou, Jinny Huang, Xusong Luo, Jun Yang

**Affiliations:** 1 Department of Plastic and Reconstructive Surgery, the Ninth People’s Hospital, Shanghai Jiaotong University School of Medicine, Shanghai, People’s Republic of China; 2 Division of Plastic Surgery, Xinjiang Korla Bazhou People’s Hospital, Xinjiang, People’s Republic of China; 3 Department of Transplantation, Johns Hopkins Hospital, Johns Hopkins University School of Medicine, Baltimore, MD USA

## Abstract

The hair follicle serves as a melanocyte reservoir for both hair and skin pigmentation. Melanocyte stem cells (MelSCs) and melanocyte progenitors reside in the bulge/sub-bulge region of the lower permanent portion of the hair follicle and play a vital role for repigmentation in vitiligo. It would be beneficial to isolate MelSCs in order to further study their function in pigmentary disorders; however, due to the lack of specific molecular surface markers, this has not yet been successfully accomplished in human hair follicles (HuHF). One potential method for MelSCs isolation is the “side population” technique, which is frequently used to isolate hematopoietic and tumor stem cells. In the present study, we decided to isolate HuHF MelSCs using “side population” to investigate their melanotic function. By analyzing mRNA expression of *TYR*, *SOX10*, and *MITF*, melanosome structure, and immunofluorescence with melanocyte-specific markers, we revealed that the SP-fraction contained MelSCs with an admixture of differentiated melanocytes. Furthermore, our *in vivo* studies indicated that differentiated SP-fraction cells, when fabricated into a *cell-chitosan/gelatin composite*, could transiently repopulate immunologically compromised mice skin to regain pigmentation. In summary, the SP technique is capable of isolating HuHF MelSCs that can potentially be used to repopulate skin for pigmentation.

## Introduction

The hair follicle is a complex mini-organ that acts as a depot for stem cells, precursors, transiently amplifying cells (TACs), and differentiated cells. The melanocyte (MC) compartment in the upper portion of the hair follicle (permanent portion) provides a reservoir for the repigmentation of the epidermis and for the cyclic formation of new anagen hair bulbs (transient portion)^1^. Melanogenesis is a multistep process starting with the enzymatic oxidation of L-tyrosine catalyzed by tyrosinase (TYR)^2^, which is strictly coupled to the growth phase (anagen) of the haircycle^1^. The end-products of L-tyrosine oxidation, the melanins, are polymorphous and multifunctional biopolymers represented by eumelanin, pheomelanin, neuromelanin, and mixed melanin pigments^3^. They are responsible for the varying hair colors among different ethnicities. The key to understanding the mechanism of cyclic melanin production is the melanocyte stem cell (MelSCs) population. The MelSCs are amelanotic and highly self-maintaining cells residing in the bulge/sub-bulge area of the lower permanent portion (LPP) of the hair follicle^4,5^. The MelSC niche^6^ is located in the “bulge” beside the muscle arrector pili attachment site and remains there throughout the hair cycles^7^. The bulge region was found to be a relatively immune-privileged site, protecting not only the hair follicle epithelial stem cell reservoir^8^ but also the MelSCs^9,10^ from autoimmune attacks. During the phase transition from telogen to anagen, intrinsic activation of MelSCs leads to the regeneration of MC progenies which in turn generate differentiated MCs that produce melanin pigment. Study shows that wounding-induced folliculogenesis in mice forms the new hair follicles with the re-establishment of the hair follicle stem cell (HFSC) population and appendages but without the re-establishment of MelSCs, causing loss of color in the regenerated hair^7^. Moreover, the presence of gray hair in vitiligious lesions has been considered as a sign of MelSCs depletion in the hair follcile^11,12^. These findings indicate that MelSCs are of high importance and are the real melanocyte reservoir for repigmentation in the hair shaft and skin epidermis^13^.

Vitiligo is the most common skin pigmentary disorder in the world^14^. It is characterized by progressive autoimmune destruction of mature epidermal melanocytes and causes psychological and social stigma in patients^15^. MelSCs were found capable of re-populating melanocytes in vitiligo lesions to regain pigmentation^16–18^. In vitiligo, although the lesional epidermis is eventually completely devoid of MCs, the pigmentation of terminal hairs is usually preserved, which suggests the presence of an intact bulge MC reservoir in depigmented vitiligo skin that is spared from the effects of the immune attack^19^. In clinical practice, non-cultured extracted human hair follicle outer root sheath cell suspension (NCORSHFS) has been proven to be effective in treating stable vitiligo patches^20–23^. However, methods like NCORSHFS that involve repigmentation through cellular grafting are generally used for small depigmentary lesions. They are not appropriate for more massive lesions due to the lack of sufficient donor hair sources, especially if the patient also suffers from hair loss caused by other diseases such as alopecia areta or scalp burn scar. These disadvantages impede the broader application of NCORSHFS and prompt us to exploit a more economical way to efficiently obtain favorable MCs from a small number of hair follicles to treat vitiligo, such as *in vitro* isolation of MelSCs and cell propagation with extended cell culture.

Unfortunately, the *in vitro* isolation of MelSCs has not yet been successfully accomplished in adult human hair follicles (HuHF)^13,24–26^, although they have already been identified *in situ*^4^ and isolated *in vitro*^25–27^ in rodents. This is mainly due to the lack of specific MelSC molecular surface markers and inapplicability of transgenic approaches. In the present study, the authors decided to explore isolating MelSCs from HuHF using the “side population” (SP) technique, which is widely used in sorting hematopoietic stem cells^28^ and tumor stem cells^29–31^. The SP technique uses the differential dye-efflux capabilities of different cells to sort them with fluorescent flow cytometry. With this technique, no molecular markers are needed. After sorting, the authors investigated the pigmentary functions of the SP fraction-derived differentiated cells. This could potentially help broaden the application of using MelSCs and cultured follicular melanocytes to treat skin pigmentary disorders.

## Results

### MelSCs *in vitro* isolation

**Figure 1:**
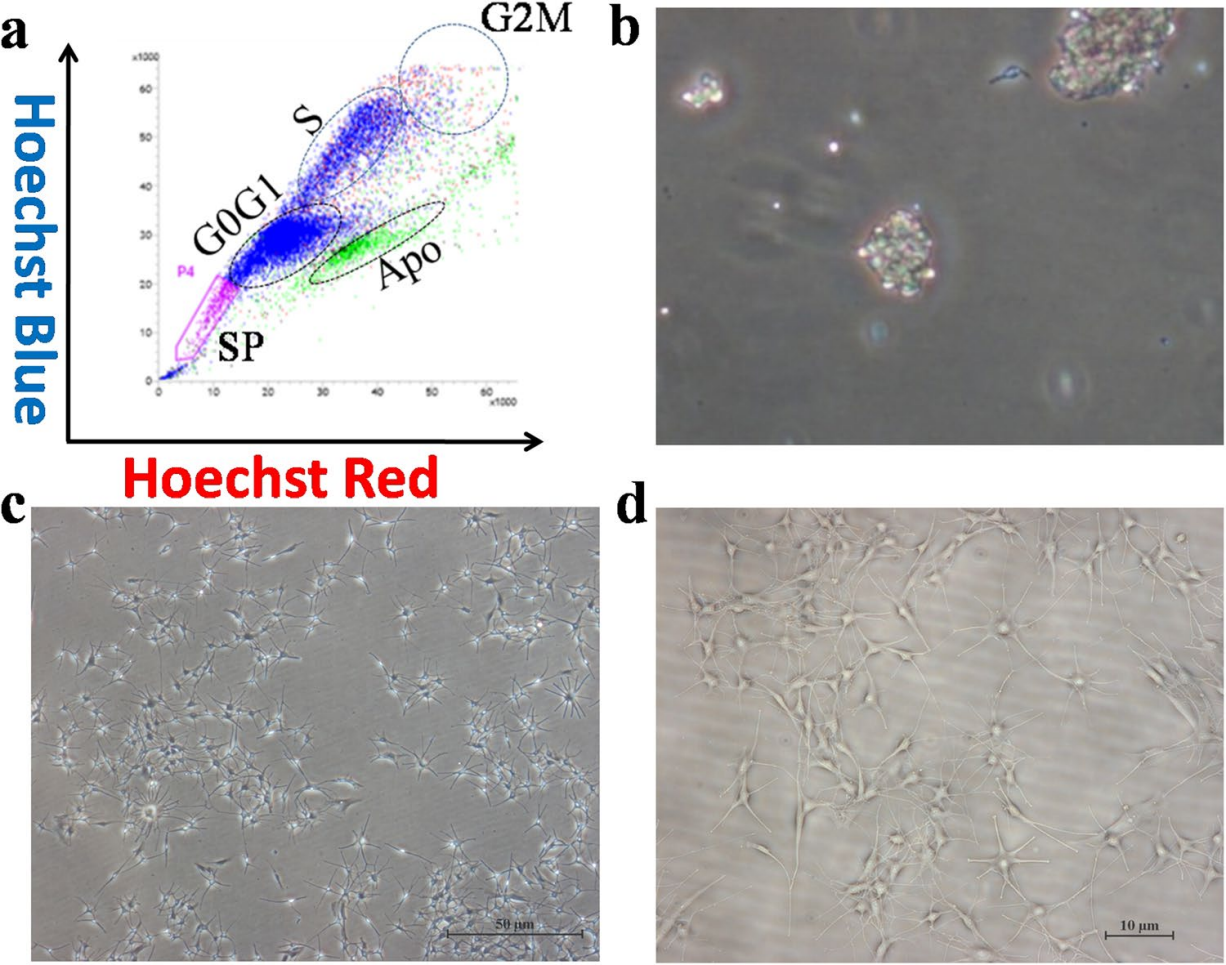
SP-gating strategy and cell cultivation. (**a**) Dissociated HuHF cells were separated into SP-p0 and different phases of the cell cycle (G0/G1, S, and G2/M) using SP technique. (**b**) Colony-forming,unit-like morphology in the primary culture of SP-p0. (**c**) SP-p4 cells displayed slender, multipolar cell protrusions with strong refractivity (4x). (**d**) SP-p4 cells displayed the typical melanocyte morphology (10x). SP: side population. G0-G2: cell cycle phases. Apo: apoptosis.

In order to isolate MelSCs *in vitro*, we sorted the dissociated HuHF using the SP technique. This assay is based on the capability of SP cells to better efflux the DNA-binding dye, Hoechst 33342^28^, or mitochondrial-binding dye, Rhodamine123^32^ because they have a higher number of ATP-binding cassette (ABC) transporter proteins expressed within the cell matrix. These dyes will emit fluorescence when excited by a UV laser at 350 nm that can be detected in both the “Hoechst Red” (630–650 nmwavelength, long-pass filter) and the “Hoechst Blue” (405–450 nm wavelength, band-pass filter) channels. The fluorescent signal can be used to visualize cells in a specific phase of the cell cycle by indicating the DNA content per cell. The “Hoechst Red” channel is more sensitive to changes in dye concentration, so SP-fraction cells (SP-p0) will emerge as a distinct dim “tail” extending first on the left side of G0/G1 cells toward the lower “Hoechst Blue” signal and retain low fluorescence retention^33,34^. As expected, our results revealed a spindly-like “tail” emerging toward the lower-left corner in approximately 1.5% of the entire live cell population, reflecting the lower dye-content and higher efflux ability (SP phenotype). There was a clear separation between different phases of cell cycle as well (Fig. 1a). The SP-p0 cells were largely lost upon inhibition of the ABC transporter activity by verapamil (see Fig. S1), a typical flow feature in SP. In the primary culture, the SP-p0 fraction was identified as colony-like cells with a spherical shape (Fig. 1b). However, the morphology of the predominant cells became more slender and spindle-like with multiple protrusions when extended to the fourth passage (SP-p4). These cells had strong refractivity (Fig. 1c) with typical melanocyte-like morphology when viewed under higher magnification fields (Fig. 1d).

### Melanocyte lineage analysis

**Figure 2:**
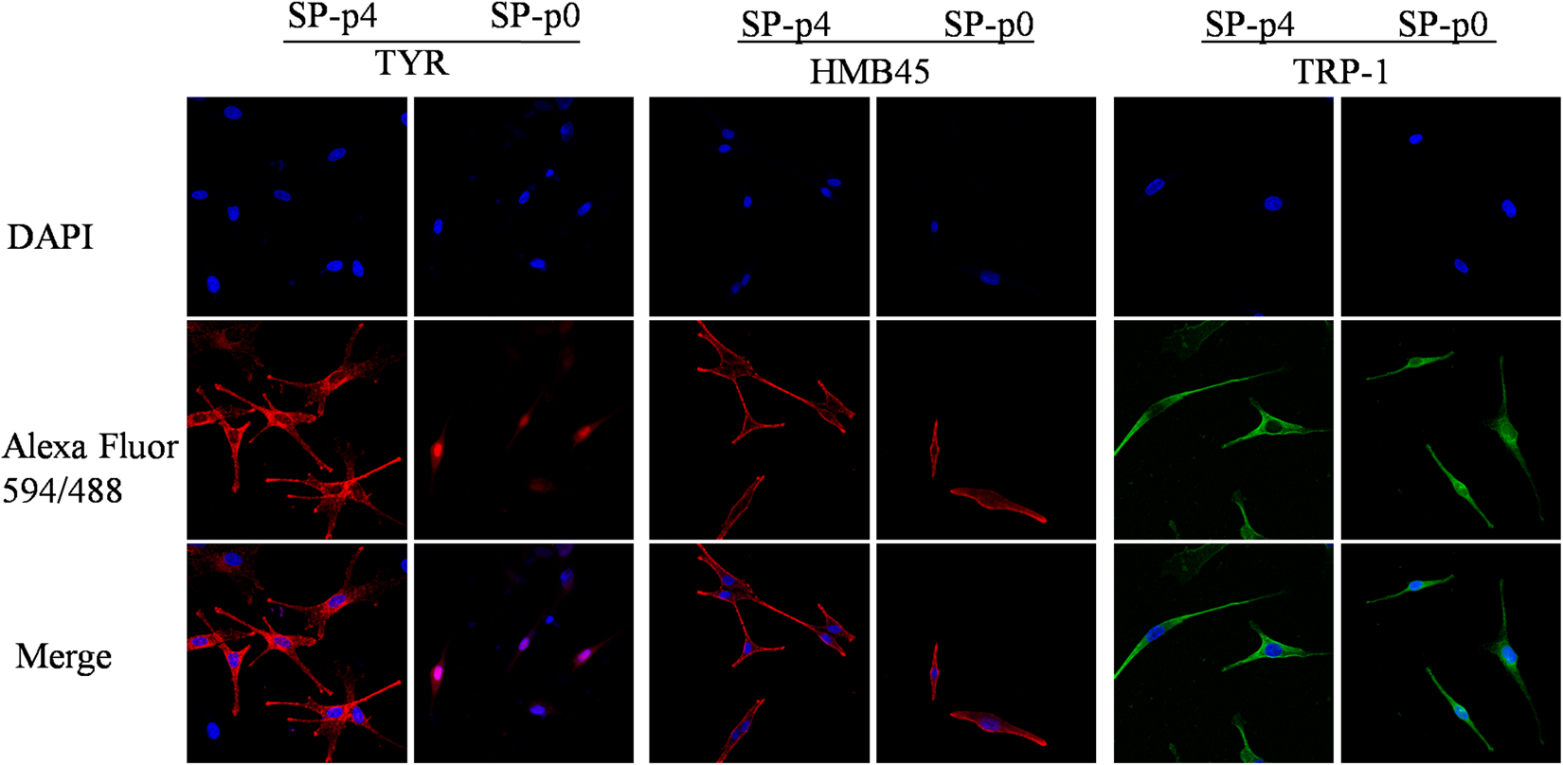
Confocal microscope images of IF in SP-p0 and SP-p4 cells (45x). Although the cells had different morphologies, both were stained positive with HMB45, TYR and TRP-1. Nuclei were counterstained blue with DAPI.

In order to characterize the side population of dissociated hair follicles for melanogenic potential, we imaged and performed immunofluorescent (IF) staining both in SP-p0 and SP-p4 cells. Melanogenic-related human melanoma black 45 (HMB45), tyrosinase (TYR), and tyrosinase-related protein 1(TRP-1) are melanocyte-specific molecular markers located in the cytoplasm. HMB45 recognizes the luminal fragment of gp100, a melanosomal protein. TYR is a rate-limiting enzyme which is necessary for melanogenesis. TRP-1 is another important enzyme involved in melanin biosynthesis. According to the results, SP-p4 cell bodies were thin and spindly-like in shape with multiple protrusions, while SP cell bodies were smaller with dipolar protrusions. Intriguingly, both were immunostained positive for HMB45, TYR and TRP-1 (Fig. 2). The nuclei were counterstained blue with DAPI. SP-p4 cells possessed the melanocyte lineage identity similar to mature p4 MCs cultured directly from dissociated HuHF w/o SP sorting (HuHF-p4) (see Fig. S2). The fact that SP cells were stained positive with melanogenic-related markers indicates either the admixture of TACs and mature MCs in the SP population, or cell differentiation occurred during the preparation process for IF when cells were incubated overnight on the coverslips.

### Differentiation analysis of SP

**Figure 3:**
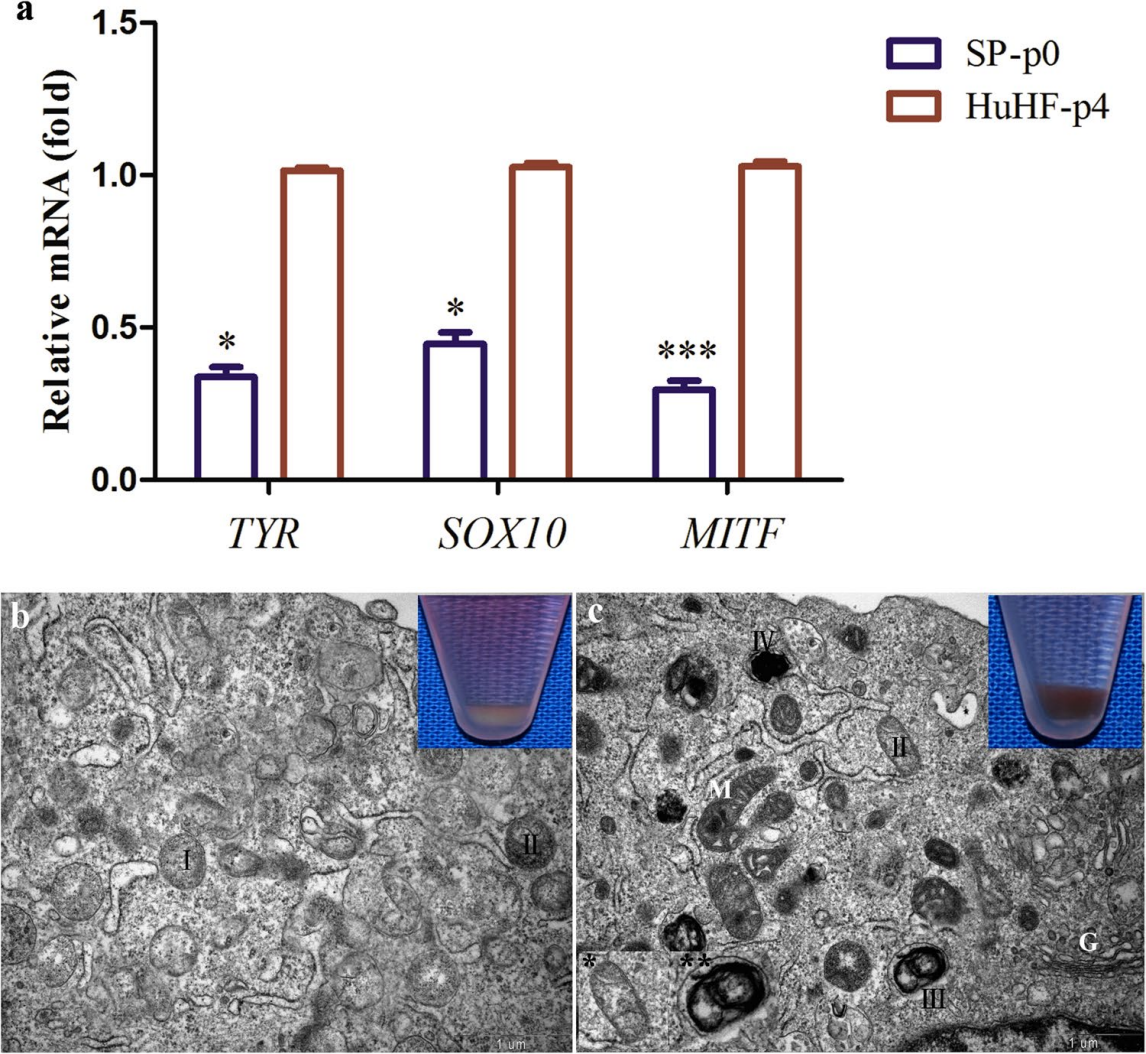
Melanogenic-related mRNA expression was significantly down-regulated in *TYR*, *SOX10* (*P < 0.05) and MITF (***P < 0.001) when comparing SP-p0 to HuHF-p4 (**a**). Macroscopically, the cell pellet color in SP-p0 was much lighter than that in HuHF-p4 (**b,c**). Pheomelanosomes in SP-p0 were predominately at stage I with no remarkable presence of mitochondria, RER or Golgi apparatus (**b**). Pheomelanosomes in HuHF-p4 were much more differentiated and predominately at stage III and stage IV. They display a gray/black cell pellet color with obvious cytoplasmic organelles. M: mitochondria. G: Golgi apparatus. Scale bar: 1 μm.

In order to assess whether the SP-p0 cells were truly less-differentiated MCs, SP-p0 and HuHF-p4 were analyzed using transmission electron microscopy (TEM) and reverse transcription-quantitative polymerase chain reaction (RT-qPCR). The structure of the melanosome highly correlates with the type of melanin produced^3,35^. The eumelanosome produces black pigment eumelanin and is usually elliptical with a fibrillar matrix. The pheomelanosome produces reddish-yellow pheomelanin and contains a vesiculoglobular matrix with a predominant spherical contour. Eumelanosomes are only capable of producing and depositing melaninat stage III and IV^36^ while in pheomelanosomes, melaninis already formed at stageII^3^. There is no melanin production at stage I in either melanosome. During melanogenesis, the enzymatic and structural elements in the cytoplasm are chronologically orchestrated^37^. Additionally, melanogenic-related genes such as *TYR*, *Sry-related HMG-box-10* (*SOX10*), and *microphthalmia-associated transcription factor* (*MITF*) are dynamically up-regulated when differentiating towards maturation^13^ because certain enzymatic proteins need to be transcripted sequentially, especially TYR, a rate-limiting enzyme for melanin synthesis. Compared with HuHF-p4, mRNA levels of *TYR*, *SOX10* and *MITF* were significantly down-regulated in SP-p0 (P < 0.05) (Fig. 3a). Various cytoplasmic organelles like mitochondria, rough endoplasmic reticulum (RER), Golgi apparatus and melanosomes at four distinct stages can be found using TEM. RER and Golgi apparatus activities are closely related with the assembling and secretion of enzymatic proteins, which is ATP-powered by mitochondria. Judging from the morphologies and matrix types displayed in the experimental results, “pheomelanosomes” rather than “eumelanosomes” were predominantly present in both SP-p0 and HuHF-p4 TEM photographs. It is worth mentioning that in black hair donors, the eumelanogenic and pheomelanogenic melanosomes can coexist in the same melanocyte^38,39^ and some atypical melanosomes may be present^40^. Pheomelanin-containing melanosomes with a eumelanogenic ultrastructure (*) and melanosomes with mixed vesicular and fibrillar matrices (**) were observed (Fig. 3c) in the HuHF-p4. In SP-p0, cell pellets were white-colored. The majority of the pheomelanosomes were at stage I, and the rest were at stage II. There were hardly any mitochondria, RER and Golgi apparatus found in the cytoplasm (Fig. 3b). However, in HuHF-p4, thecolor of the cell pellets was gray or black. Pheomelanosomes and atypical melanosomes were predominately present at stage III and stage IV. The obvious distribution of mitochondria and Golgi in the cytoplasm, along with the presence of gray/black cell pellets, indicates active melanin synthesis (Fig. 3c).

### Fabrication of *cell-chitosan/gelatin composite*

**Figure 4:**
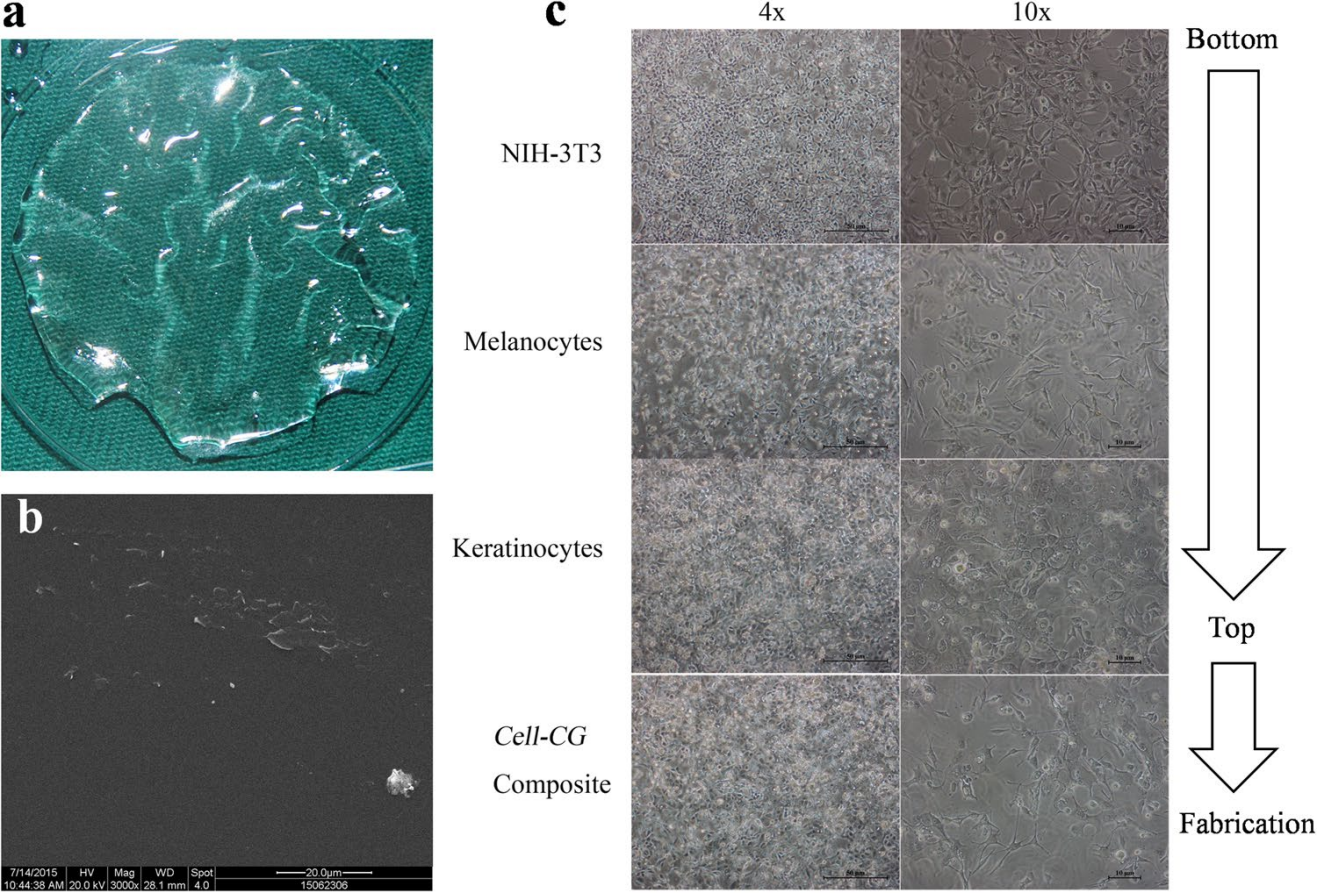
(**a**) Transparent physical form of C/G matrix in the culture medium. (**b**) 2-dimensional architecture examined by SEM. (**c**) Photographs of *Cell-C/G composite* under phase contrast microscope. NIH-3T3, MCs, KCs, and spatial cell-cell interactions were revealed from bottom to top with minor adjustments in the microscope focal length.

In order to fabricate a *cell-chitosan/gelatin composite* for *in vivo* use, we employed a commonly used chitosan-gelatin (C/G) membrane^41^ which was previously described by our research group^42^. Chitosan shares a similar molecular structure with glycosaminoglycans (GAGs), and the gelatinis composed of denatured collagen with high amino acid content. C/G composites mimic the natural components of the extracellular matrix (ECM). However, increased proportions of gelatin in the C/G blend are correlated with increased cell adhesion but decreased mechanical properties^41^ due to changes in hydrophilicity. To achieve favorable mechanical properties that facilitate cell transfer, a C70: G30 (a weight ratio of 7:3) matrix was blended. The ratio was C75: G25 in Cheng’s research^41^, which exhibited the same prosperities. This manufactured C/G matrix was a transparent, insoluble membrane-like matrix (Fig. 4a) with strong tensile strength^41,42^. Scanning electron microscopy (SEM) indicated that this blended matrix had a 2-dimensional surface structure examined at 25.0 kGy (Fig. 4b). This matrix was tested favorable for MC but not keratinocyte (KCs) adhesion (see Fig. S3). In order to improve KCs adhesion and cell interaction, NIH-3T3 feeder cells were seeded to the C/G matrix surface prior to the MCs and KCs. MCs adhered to the C/G matrix faster and easier than KCs (data not shown). Sequentially within the dish from bottom (distal to eyepiece of microscope) to top (proximal to eyepiece of microscope), NIH-3T3 feeder cells, multipolar MCs and “cobblestone-like” KCs were, identified respectively (Fig. 4c). These three kinds of cells were distributed within each other’s interspace and were inclined to form physiological cell-cell interactions. When the mixed cells reached 80–90% confluence, they were ready to be transferred to repair the skin lesion.

### *In vivo* pigmentation and immunohistochemistry

**Figure 5:**
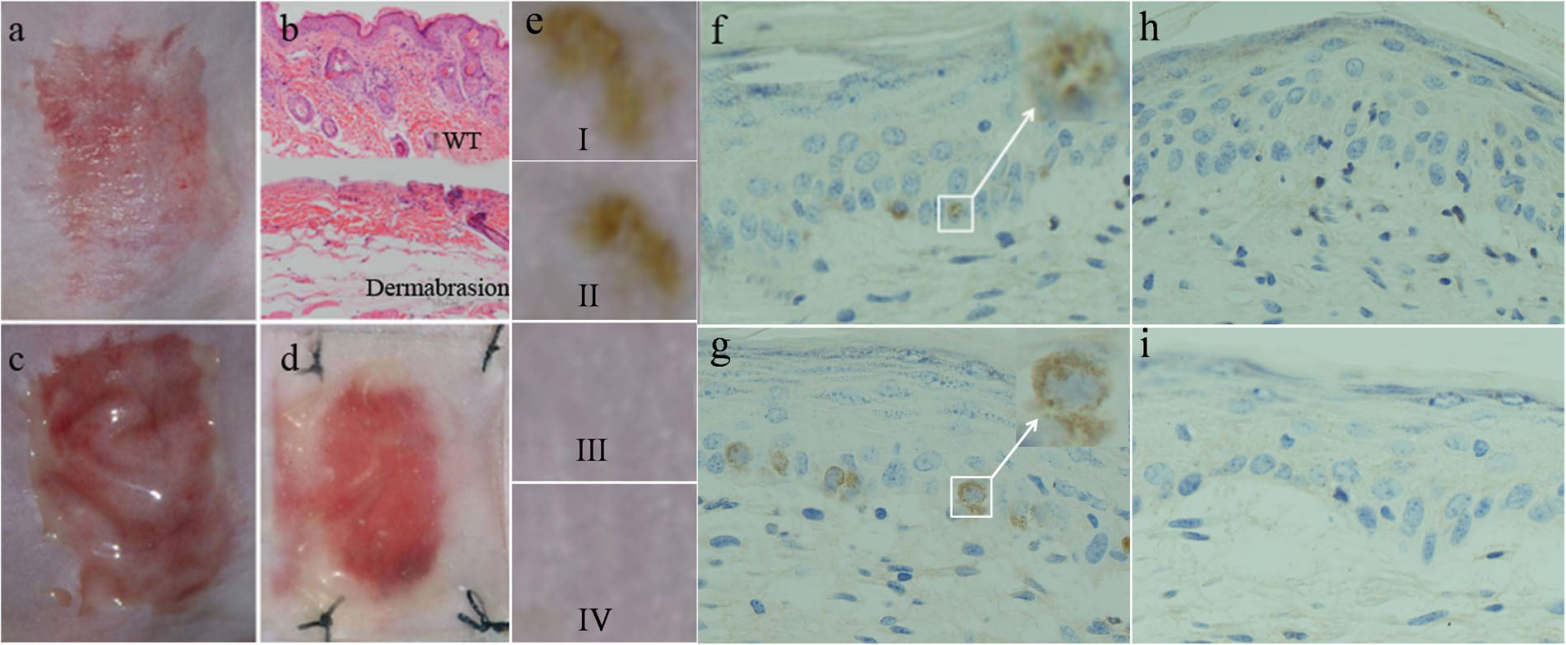
*In vivo* application *of cell-C/G composite* and skin IHC staining with TYR. (**a**) Dermabrasion of split-thickness skin defect. (**b**) H&E staining for the lesion and WT skin. (**c**) *Cell-C/G composite* application in the mouse dorsal skin. (**d**) Silicone sheet coverage. (**e**) Pigmentation in four groups. IHC anti-human TYR staining results in group I (**f**), group II (**g**), group III (**h**) and group IV (**i**) (4x).

To assess its capability to repopulate skin for pigmentation, the *cell-C/G composite* was applied to dermabraded wounds. Skin pigmentation was monitored weekly. Biopsies for immunohistochemistry (IHC) were processed at the onset of pigmentation or at 8 weeks post-dermabrasion (the terminal time point) if no obvious pigmented dot was found. The results revealed that the split-thickness skin defect was created (Fig. 5a). This was further confirmed by comparing hematoxylin-eosin (H&E) staining with that in wild type (WT) mouse skin (Fig. 5b). The *cell-C/G composite* was applied in the skin lesion (Fig. 5c) and secured with silicone gel (Fig. 5d). Within approximately 2–4 weeks, a small number of tiny pigmented dots with the average size of 0.02 cm^2^ occurred in groups I (K: M = 1:5 *cell-C/G composite*) and II (K: M = 1:10 *cell-C/G composite*) in each animal (Fig. 5e), while no pigmentation was observed in groups III (*cell-C/G composite* w/o MCs) and IV (C/G matrix w/o cell) (Fig. 5e). These pigmented dots had diameters of less than 0.5 mm and were randomly located. Skin biopsies from groups I (Fig. 5f) and II (Fig. 5g) stained positive for anti-human TYR while those from groups III (Fig. 5h) and IV (Fig. 5i) stained negative. IHC staining results with HMB45 were consistent with TYR (see Fig. S4). IHC analysis revealed that MCs included in the *cell-C/G composite* were responsible for repopulating the skin lesions for pigmentation. However, the brown/yellowish color of pigmented dots gradually faded away and ultimately vanished within 2 months post-occurrence (ranging from 4 to 8 weeks, average 6.1 weeks, data not shown), which implies that it is a transient pigmentation.

## Discussion

MelSCs reside in the bulge/sub-bulge area of the LPP of the hair follicles. This area serves as a melanocyte reservoir for pigmentation in the hair matrix and skin epidermis^13^. Loss of MelSCs causes hair graying^11^ and vitiligo^17^ when functional MCs run out. With regard to vitiligo, researchers deem that the lack of progress in vitiligo repigmentation is largely due to an incomplete understanding of MelSC activation in the hair follicle^17^. Previous evidences showed that MelSCs and their progenies, melanocyte progenitors (MPs), play a vital role in the melanocyte homeostasis and repigmentation^15,16,43^. The MPs are presumed to be the amelanotic melanocytes (AMMCs)^44–47,48^. They are abundant in the LPP of HuHF^43,48^, which is the same location as the MelSCs/MPs niche. The absence of AMMCs in some black-colored hair shows similar distribution patterns and cellular behaviors as mouse MelScs^49^ and may explain treatment failure in some vitiligo patients^50^. Studies on AMMCs revealed that they migrated to the interfollicular epidermis, leading to the “follicular repigmentation” that is clinically visible in repigmented vitiligo lesions^43^. Since human MelSCs/MPs are very important physiologically and pathologically, it would be beneficial to isolate them *in vit*ro. It is important to note that the hair plucking preparation in the present experiment is used to keep the “bulge” structure intact *in situ* along with the plucked hair. This is very different from other studies that only focus on the outer root sheath (ORS) cell suspension^18–21,47,48^ because the ORS is not the site where MelSCs and MPs reside. In this experiment, we treated the scalp with dispase in order to evenly loosen the subcutaneous tissue. This will ease the complete-hair plucking process while keeping the bulge region attached. According to our experiment results, the SP sorting technique can be used for isolating an impure fraction of MelSCs if no molecular markers are available. These SP fraction cells contain some stage II pheomelanosomes and expressed low levels of melanogenic-related genes like *TYR* and *TRP-1*. Furthermore, they stained positive for melanocyte-specific markers despite their distinct morphologies and dipolar protrusions. Interestingly, their morphologies are quite similar to the melanoblasts cultured *in vitro* in previous works^51^. IF staining, however, might not be objective enough to firmly conclude that these cells are not truly MelSCs/MPs. The colony-forming unit indirectly indicated the pre-existence of MelSCs or MPs. Moreover, SP phenotype is not exclusive to stem cells^29,52–54^ and can also occur in differentiated tissues^55,56^. Based on the aforementioned evidence, we speculate that SP fraction contains MelSCs/MPs with an admixture of TACs and MCs. To better investigate the purification and identification efficacy of SP isolation, multiple molecular markers and flow cytometry should be used in future studies.

To better understand whether SP fraction cells were able to produce melanin in the skin, we differentiated and expanded SP cells into more fully mature MCs. It has not been successful to culture HuHF-derived MCs *in vitro* for many years until a breakthrough was made by Tobin^46^ *et al*. in 1995. Improved approaches were further developed^24,57,58^ afterwards. Melanin granules are synthesized in the melanosomes and transferred into cortical and medulla KCs, resulting in the formation of pigmented hair shafts and skin. In the adult HuHF, pigmentation is attributed to precise sequential interactions between follicular MCs, matrix KCs, and dermal papilla (DP) cells^1^. DP cells are able to induce *de novo* hair-follicle growth not only in rodent^59^ but also in human skin^60^. KCs interact with MCs by cell-cell adhesion at a ratio of 1:10 in the skin^61,62^ and at different cell ratios in the hair depending on the current developmental stage^2,63,64^. KCs can secret growth factors such as stem cell factor (SCF, or KitL, the ligand of c-Kit) and basic fibroblast growth factor (bFGF)^62^. MCs express c-Kit, which is the main tyrosine kinase receptor. SCF/c-kit signaling is required for cyclic regeneration of the hair pigmentation unit^65^. DP fibroblasts can secrete the proteins Kitl, EDN3, and FGF2, which are vital for the growth and proliferation of MCs^26^. In our study, the HuHF-derived KCs and NIH-3T3 feeder cell line (mouse immortal embryo-derived fibroblasts) were used for the fabrication of *cell-C/G composite*. The repopulation of MCs occurred through cell-cell interactions similar to the ones described above, and the melanotic function of MCs/MPs resulted in melanin production in the skin. Intriguingly, the pigmentation only lasted for approximately two months. The most probable reason for this phenomenon may be due to the transient presence of melanogenic MCs along with the lack of the MelSCs niche in the *cell-C/G composite in vivo*. The MCs undergo several cycles of multiplication and undergo apoptosis w/o sustainable SCF expression. This may be because murine KCs *in situ* might lack expression of Scf, causing them to be incapable of maintaining the MCs. Furthermore, the phenotype of this mouse strain causes hair hypogenesis, especially in the stratum corneum layer. This increases cornification rate and makes the skin more susceptible to mechanical injury^66,67^. Loss of KCs would thus lead to the vanishing pigmentation.

The material porosity of scaffolds is an important parameter that needs to be taken into consideration. Chitosan has a porous microarchitecture. However, the admixture of gelatin resulted in the reduction of pore size^68^, and more evidently decreased pore size was found with higher gelatin content^69^. In our study, the C70: G30 transformed the 3-dimemsional chitosan into a 2-dimensional C/G matrix. Although the 3-dimensional scaffold is good for reshaping and reconstruction, the 2-dimensional structure is more beneficial for cell transfer and is regarded as a promising candidate material for cell therapy^41^. The *Cell-C/G composite* was fabricated with an ingredient of this 2-dimensional C/G matrix. Consistent with our previous finding^42^, the C/G matrix facilitated MCs repopulation in dermabraded skin lesions. The transient presence of pigmentation proves that cultured follicular MCs derived from SP isolation can differentiate and expand to repopulate the lesional skin. In future studies, protocols to isolate pure MelSCs/MPs and determine their optimal culture conditions need to be established for further MelSCs/MPs research in vitiligo.

## Conclusions

This study used the side population technique to isolate HuHF-derived MelSCs/MPs *in vitro*. The result was an admixture of transiently amplifying cells and mature melanocytes in the SP fraction cells. The *cell-C/G composite* fabricated with a 2-dimensional C/G matrix and the SP-derived melanocytes was capable of repopulating melanocytes in the lesional skin and starting the transient presence of pigmentation. This provides insight into using cultured human follicular melanocytes to potentially treat skin pigmentary disorders for the future research.

## Materials and Methods

### Patients

This work was approved by the institutional review board (IRB) of Shanghai Ninth People’s Hospital affiliated with Shanghai Jiaotong University School of Medicine and conducted in accordance with its ethical standards, as well as the Helsinki declaration. Human scalp was obtained from patients who received face lift surgeries with informed consent. These patients were with non-alopecia and non-declared disorders of pigmentation.

### Animals and anesthesia

The experiment was approved by the Institutional Animal Care and Use Committee (IACUC) of Shanghai Jiaotong University School of Medicine. Immunologically compromised male nude mice (Balb/c-origin, *Foxn1*^*nu*^*/Foxn1*^*nu*^phenotype, 8 weeks old) were purchased from the Sino-British SIPPR/BK Lab Animal LTD., Shanghai, People’s Republic of China, provided *ad libitum* access to food and water, and maintained in a 12-h light–dark cycle room. Animals received care in compliance with the *Guide for the Care and Use of Laboratory Animals* published by the National Institutes of Health. 5% chloral hydrate was used for general anesthesia at a dosage of 0.005 ml/g IP. Mice were euthanized with an overdose of 10% chloral hydrate followed by cervical dislocation.

### Manufacture of C/G matrix

C/G matrix was fabricated according to our previously published work^42^ with slight modification. Briefly, chitosan (C3646, Sigma,St. Louis, MO, USA) and gelatin (G1890, Sigma, St. Louis, MO, USA) were blended at a weight ratio of 7:3 and were dissolved in 0.5 M acetic acid and distilled water. After being completely stirred using a magnetic bar and sonicated at 60 °C for 2 h, this homogeneous mixture was poured into a Petri dish (d = 10 cm) with a thickness of approximately 0.5 mm, desiccated at 37 °C for 24 h, and washed copiously using MilliQ water (Millipore, Billerica,MA, USA). All samples were lyophilized at −80 °C to obtain C/G matrix and finally sterilized using γ-irradiation at 25 kGy.

### C/G morphology analysis

The morphology of C/G matrix was examined using a scanning electron microscope (SEM, Philips/FEI QUANTA 200, Hillsboro, OR, USA). For this purpose, samples were dried using a series of increasing concentrations of ethanol followed by a brief vacuum drying. They were then sputter-coated with gold-palladium and imaged at an acceleration voltage of 20–80 kV.

### Dissociated HuHF cells

Dissociated HuHF was prepared step by step as previously described^58^ with some modifications: the scalp was sliced, disinfected, and digested using 0.2% dispase at 4 °C overnight to detach the epidermal layer from the dermis. The epidermis was removed using a pair of micro-forceps under an optical microscope equipped with a Leica DFC420 Ccamera. Hair follicles were plucked out in the direction of hair growth. They appear as a transparent and narrow gelatinous envelope around the proximal (root) part of the hair shaft. The hair bulb was removed by cutting the proximal portion (the lower 20% in length) to decrease the possibility of fibroblasts contamination in coming adhesive cell culture. Hair follicles were collected and digested with 0.04%-trypsin/0.03%-EDTA trypsin (C-41220, Promocell, Heidelberg, Germany) for 10 min at 37 °C on a bench rocker. Trypsinization was terminated using 20 ml of trypsin neutralization solution (TNS, C-41220, Promocell, Heidelberg, Germany). Cells were filtered through a 70-µm strainer (352340, Corning, NY, USA) and centrifuged at 20 °C with 220 × g for 5 min.

### “Side population”

SP protocol was conducted as previously described^12^ with slight modifications. According to the manufacturer’s protocol, HuHF was treated using trypsin (C-41220, Promocell, Heidelberg, Germany), filtered through 70-µm strainers (352340, Corning, NY, USA), pelleted by centrifugation, and resuspended in prewarmed Hank’s balanced salt solution (HBSS) containing 2% FBS and 1 mM HEPES. Cells were then labeled with Hoechst 33342 dye (B2261, Sigma, St. Louis, MO, USA) at a final concentration of 5 μg/ml and incubated for 120 min at 37 °C, either alone or with verapamil (V4629-1G, Sigma, St. Louis, MO,USA) with a concentration of 50 μM. Cells were then counterstained with 1 µg/ml propidium iodide (PI) to label dead cells. 1 × 10^6^ viable cells/ml were analyzed and sorted by an Influx^TM^ sorter (BD, NJ, USA). Hoechst dye was excited with a UV laser at 350 nm, and its fluorescence was measured at wavelengths using 650 nm (Hoechst Red channel) and 450 nm (Hoechst Blue channel) optical filters. SP-p0 cells were cultivated in DLMa melanocyte medium (LL-0039, Cellsystems, Kirkland, WA,USA) in an incubator at 37 °C under hypoxia condition (5% O_2_, 5% CO_2_). Culture medium was changed every 3–4 days. Adherent cells were expanded and extended to the fourth passage (SP-p4) with melanocyte growth medium (M-GM, C-24010, Promocell, Heidelberg Germany) in an incubator at 37 °C under normoxic conditions (5% CO_2_, 95% air).

### Immunofluorescence

SP-p0 and SP-p4 were immunostained with melanocyte-specific molecular markers. 1 × 10^2^ cells were seeded on ø 22 mm fibronectin-coated round coverslips (354088, Corning, NY, USA) which were placed in the 12-well TC-treated plates (3513, Corning, NY, USA) and incubated overnight. They were then fixed using 4% paraformaldehyde (PFA) for 15 min at 4 °C and permeabilized using 0.3% Triton X-100 for 10 min. After thorough washing with PBS, 10% goat blocking serum was applied to reduce nonspecific staining. The cells were then incubated overnight in the dark at 4 °C with melanocyte-specific antibodies HMB45 (1:100; mouse anti-human, ab787, Abcam, Cambridge, UK), TYR (1:50; mouse anti-human, ab58284, Abcam, Cambridge, UK) and TRP-1(1:100; mouse anti-human, MABC592, Millipore, Billerica, MA, USA). The next day, the cells were incubated with secondary goat anti-mouse antibodies coupled with either red fluorescence (1:1000; Alexa Fluor 594, A-11005, Invitrogen, Carlsbad, MA, USA) or green fluorescence (1:1000; Alexa Fluor488, A-11001, Invitrogen, Carlsbad, MA, USA) were incubated subsequently. DAPI (1:100; C0060, Solarbio, Beijing, China) was used for nuclear counterstaining. Slides were photographed with a confocal laser scanning microscope (LSM-710, ZEISS, Oberkochen, Germany).

### RT-qPCR

*TYR*, *SOX10 and MITF* gene expression in SP-p0 and HuHF-p4 was analyzed by RT-qPCR. RNA was extracted by Trizol to synthesize cDNA through reverse transcription. At the end point of RT-PCR, a 97-bp fragment of *TYR*, 160-bp fragment of *SOX10*, 97-bp fragment of *MITF*, and 105-bp fragment of *GAPDH* were amplified with SYBR Green Fluor qPCR Master mix (Qiagen, Hilden, Germany) following the manufacturer’s protocols on a Real-Time PCR Detection System (Applied Biosystems Instruments, Waltham, MA, USA). The forward and reverse primers used were as follows: *Tyr*, forward, 5′-GCACCCCACAAATCCTAACT-3′ and reverse, 5′-ACTCCTCCAATCGGCTACAG-3′; *Sox10*, forward, 5′-GCTGCTGAACGAAAGTGA-3′ and reverse, 5′-CCTGGGCTGGTACTTGTA-3′; *Mitf*, forward, 5′-AGACGGAGCACACTTGTTAG-3′ and reverse, 5′-ACCCCTTCA GGTAAGTTATTAAA-3′; and *GAPDH*, forward, 5′-GGGAAGGTGAAGGTCGGAGT-3′ and reverse, 5′-GGGGTCATTGATGGCAAC-3′. The thermo cycling parameters of PCR were as follows: denaturation at 95 °C for 1 min, followed by 40 cycles of denaturation at 95 °C for 15 s, and annealing at 60 °C for 60 s. Each trial sample was performed in triplicates. The expression level was determined by the 2^−ΔΔCt^ method using the housekeeping gene, *GAPDH*, as the reference.

### Melanosome analysis

The developmental stages of the melanosomes were examined by TEM as previously described^23^. Briefly, SP-p0 and HuHF-p4 cell pellets were fixed in 2.5% glutaraldehyde and 0.05 M phosphate mix buffer (pH 7.2) at 4 °C overnight, then transferred to a 1% osmium tetroxide and 0.1 Mphosphate mix buffer for 1 h, and stained with 1% uranyl acetate and 50% ethanol staining for 30 min. After being dehydrated using serial alcohol and acetone incubations, cells were embedded in Spurr’s resin. 80 nm sections were cut using an LKB V ultra microtome were stained using uranyl acetate and lead citrate, and photographed with a transmission electron microscope (Philip CM-120, Amsterdam, Holland).

### *In vitro* fabrication of cell-C/G composite

1 × 10^5^ NIH-3T3 feeder cells were seeded on the C/G matrix prior to the KCs and MCs. To arrest the further proliferation, 10% mitomycin C was added after 50–55% cell confluence. To maintain their viability, 1% FBS was added to the culture medium. 1 × 10^5^ p2 KCs (see Fig. S5) and 0.5 × 10^6^ SP-p4 (K: M = 1:5) or 1 × 10^6^ SP-p4 (K: M = 1:10) were co-cultured on C/G matrix for 1–2 weeks. 50%: 50% K-GM: M-GM culture medium was changed every 2–3 days. Cell morphologies were examined and photographed using a phase contrast microscope (Olympus, Tokyo, Japan). *Cell-C/G composite* was fabricated when cells reach 80–90% confluence and then was used to repair dermabraded skin in immunologically compromised nude mice.

### *In vivo* application of cell-C/G composite

Twenty mice were randomly assigned to four groups, and 5 mice were included in each group. 0.5 cm × 0.8 cm piece of dorsal skin were dermabraded using an automatic nail polish tool equipped with a diamond cone to create split-thickness skin defects. The skin wounds were then covered with 1 cm × 1.5 cm 1: 5 *cell-C/G composite* (group I), 1: 10 *cell-C/G composite* (group II), *cell-C/G composite* w/o melanocytes (group III), C/G matrix w/o cell (group IV) with the cell side facing downward, followed by a 2 cm × 2 cm size silicone gel sheet (Smith & Nephew, London, UK) to prevent migration of matrix and retain topical moisture. Skin color was monitored once a week until the onset of pigmentation, or 8 weeks post-dermabrasion, the terminal time point, even w/o pigmentation.

### Immunohischemistry

For immunohistochemistry, skin was biopsied at the onset of pigmentation or at 8 weeks post-dermabrasion (the terminal time point). Skin tissue was cut into 4-µm-thick paraffin sections. The tissue sections were incubated with antibodies against human HMB45 (1:100, mouse anti-human, ab787, Abcam, Cambridge, UK) and TYR (1:100, mouse anti-human, ab58284, Abcam, Cambridge, UK). The specimens were then incubated with the secondary antibody, an anti-mouse peroxidase-labeled polymer (1:50; ZSGB-BIO, Beijing, China). The images were captured with a light microscopy (1 × 71 inverted microscope; Olympus Corp., Tokyo).

### Statistical analysis

All data were calculated as mean ± standard deviation (SD). Statistical significance was calculated with Student’s t-test using SPSS 16.0 software for Windows (SPSS Inc., Chicago, IL, USA).P value < 0.05 was considered to indicate statistically significant^40^.

## Supplementary information

supplementary file
