# Supplementary material for: Melanocyte Chitosan/Gelatin Composite Fabrication with Human Outer Root Sheath-Derived Cells to Produce Pigment
Source: Sci Rep. 2019 Mar 26;9:5198. doi: 10.1038/s41598-019-41611-5 (PMC6435804; doi:10.1038/s41598-019-41611-5)
Supplement: Supplementary file 1 — supplementary file [file 41598_2019_41611_MOESM1_ESM.pdf]

Melanocyte Chitosan/Gelatin Composite Fabrication with Human Outer Root Sheath-Derived Cells to Produce Pigment

Xianyu Zhou[1+], Yan Ma[2+], Fei Liu[1], Chuan Gu[1], Xiuxia Wang[1], Huitang Xia[1], Guangdong Zhou[1], Jinny Huang[3], Xusong Luo[1*], Jun Yang[1*].

Fig. S1

50 µM verapamil was incubated with Hoechst 33342 dye for 120 min. Spindly-like SP-fraction cells were largely lost as calciphorin in the membrane of SP cells was inhibited, resulting in little Hoechst efflux detected by flow cytometer.

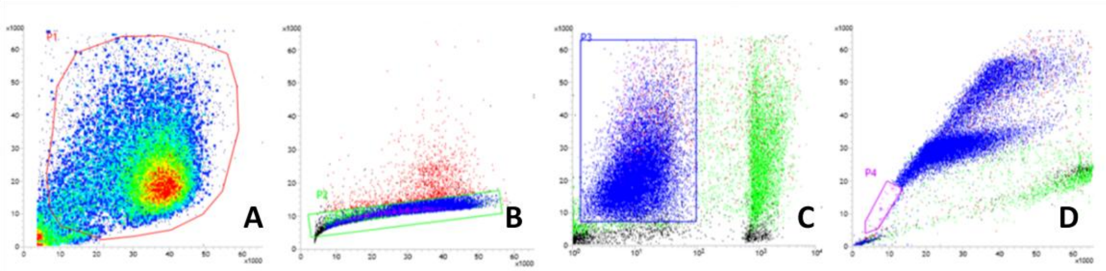

Figure S1. Verapamil inhibited the Hoechst efflux of SP fration. Cell gated w/o debris (A), singlets (B), viable cells (C) and, SP gating with verapamil incubation (D).

Fig. S2

SP-p4 melanocytes were obtained via primary and extended culture from dissociated hair follicle outer root sheath in a 5% CO_2, 37 °C incubator according to the protocol described previously by Dieckmann[58]. For immunofluorescence, 1 $\times 10^4$ cells were used and incubated for 2 hours before fixation. The rest of the procedures were performed according to the manufacture's protocol as described in the main text.

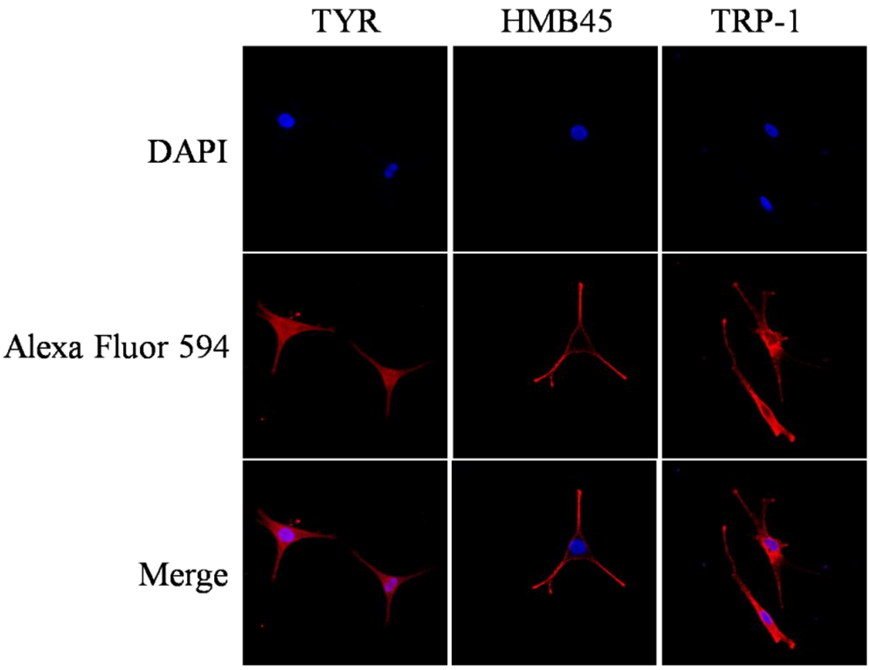

Fig. S2 HuHF-p4 melanocytes cultured from dissociated human hair follicle outer root sheath without using side population were stained positive with melanogenic-related markers, TYR, HMB45 and TRP-1. They displayed a similar morphology to SP-p4 cells.

Fig. S3

p2 keratinocytes (KCs) adhered and propagated favorably in the petri dish under regular condition, however, adhesion decreased dramatically when they were seeded on the C/G matrix. The KCs floated in the culture medium and had a spherical morphology. Therefore, although C70 : G30 matrix blend possessed appropriate physical properties, it was less biocompatible for KCs. Melanocytes (MCs) showed good adherent ability and proliferated on the C/G matrix. MCs-KCs co-culture system did not improve KC adhesive ability while MCs still adhered and expanded well. When NIH-3T3 feeder cells were seeded to coat the dish, the KCs' adhesion was substantially boosted, displaying a similar adhesive ability to being cultured ina petri dish under normal conditions.

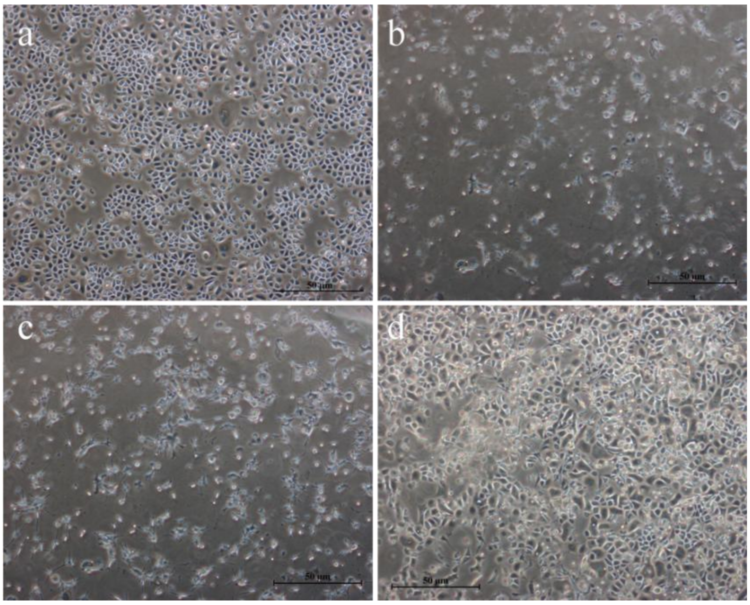

Figure S3. NIH-3T3 feeder cells facilitated KCs adhesion on the C/G matrix. (a) p2 KCs cultured in the petri dish exhibited typical “cobblestone-like” morphology. (b) p2 KCs cultured directly on the C/G matrix surface w/o NIH-3T3 presented substantially decreased adhesion, floating with spherical morphology. (c) p2 KCs and SP-p4 MCs were co-cultured on the C/G matrix surface w/o NIH-3T3. KCs demonstrated decreased adhesion similar to when cultured w/o MCs in (b). (d) p2 KCs cultured on the C/G matrix surface w/ NIH-3T3 displayed the similar adhesive ability as cultured in the petri dish of (a).

Fig. S4

For immunohistochemistry, mouse anti-human HMB45 primary monoclonal antibody was used. The procedures were performed according to the manufacture’s protocol for TYR immunohistochemical staining as described in the main text. Skin biopsies were stained positive with HMB45 in both group I and group II but negative in group III and group IV, showing the same results as those of TYR.

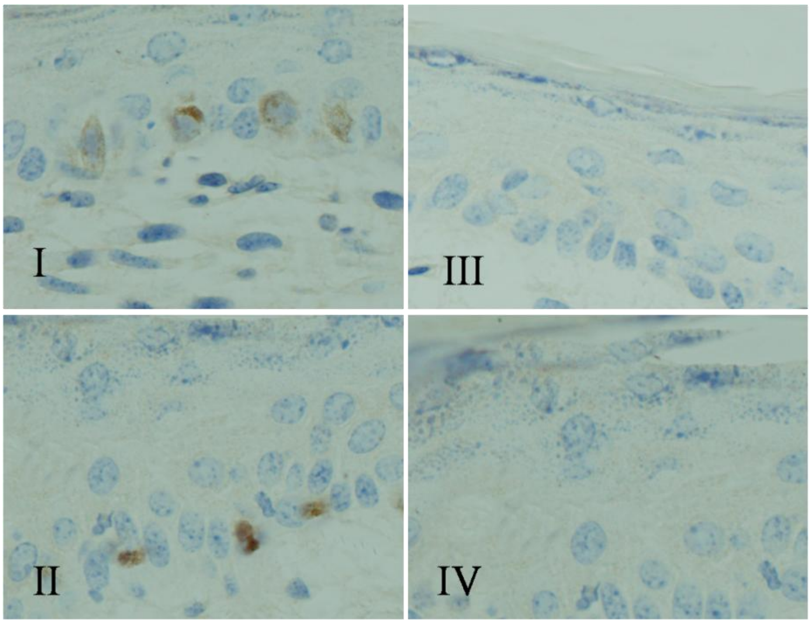

Fig. S4 Positive HMB45 immunohistochemical staining in the lesional skin in group I and group II, with negative staining in group III and group IV.

Fig. S5

Hair follicle outer root sheath-derived cell pellets were resuspended in complete keratinocyte growth medium (KGM, C-20011, Promocell, Heidelberg, Germany) and seeded with 5×106 cells per Petri dish (d=10cm) in a 37°C incubator under normoxic conditions (5% CO2, 95% air). After 8-10 days adherent culture, the keratinocytes reached ~70% confluence. These cells exhibited a typical "cobblestone-like" morphology. To extend the culture, cells were detached using 0.03%EDTA/0.04% trypsin (C-41220, Promocell, Heidelberg Germany) and sub-passaged at a ratio of 1:3. For immunofluorescence, 1×10^4 p2 keratinocytes cells were seeded on the ø 22mm fibronectin-coated round coverslips (354088,Corning, NY, USA) placed in the 12-well TC-treated plates (3513, Corning, NY, USA) and incubated overnight in a 37°C incubator under hypoxic conditions. Adherent cells were then fixed using 4% paraformaldehyde (PFA) for 15 min at 4°C and permeabilized with 0.3% Triton X-100 for 10 min. After thorough washing with PBS, 10% goat blocking serum was applied and cells were then incubated overnight in the dark at 4°C with anti-pan cytokeratin (1:100, mouse anti-human ab86734, Abcam, Cambridge, UK). The next day,

cells were incubated with secondary goat anti-mouse antibody coupled with red fluorescence (1:1000; Alexa Fluor 594, A-11005, Invitrogen, Carlsbad, MA, USA). DAPI (1:100; C0060, Solarbio, Beijing, China) was used for nuclear counterstaining. Slides were photographed with a confocal laser scanning microscope (LSM-710, ZEISS, Oberkochen, Germany).

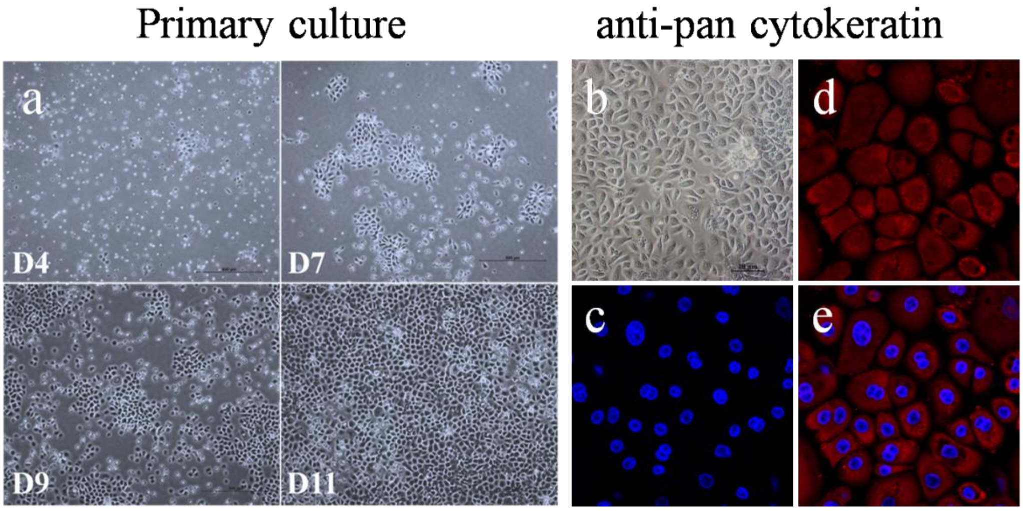

Figure S5 Cultivation and identification of ORS-derived keratinocytes. (a) Primary culture of hair follicle outer root sheath-derived keratinocytes. (b) p2 sub-passage displaying typical “cobblestone-like” morphology. (c-e) Positive immunofluorescent staining with anti-pan cytokeratin.
